# LONGITUDINAL EVIDENCE FOR DISCREPANT CHANGES IN NEGATIVE AFFECT REACTIVITY AND BLOOD PRESSURE REACTIVITY WITH AGE

**DOI:** 10.1093/geroni/igz038.2290

**Published:** 2019-11-08

**Authors:** Rachel E Koffer, Thomas W Kamarck

**Affiliations:** University of Pittsburgh, Pittsburgh, Pennsylvania, United States

## Abstract

Both affective and blood pressure (BP) reactivity are associated with long term risk of chronic disease and mortality. Thus, understanding age-related changes in negative affect and BP responses to everyday demands is vital for promoting healthy aging. However, few studies have examined both psychological and BP reactivity simultaneously, which would provide more comprehensive understanding of regulatory processes at play. For the present study, 232 adults aged 50-70 years were assessed at baseline and 6 years later with ambulatory BP monitoring and momentary electronic diaries. Reactivity coefficients were output from multilevel models and used to test changes in negative affective and ambulatory BP reactivity to task demand, longitudinally. Results indicate that both systolic and diastolic BP reactivity increase with age, while negative affect reactivity does not change with age. Results are discussed in the context of life course theories of role strain and role changes and socioemotional theories of aging.

